# Epicenter mapping of cortical morphological dedifferentiation in type 2 diabetes mellitus using morphometric inverse divergence

**DOI:** 10.3389/fnagi.2026.1884185

**Published:** 2026-08-24

**Authors:** Taipeng Zeng, Liyuan Fu, Zhifeng Huang, Minghui Mao, Fang Chen, Hao Huang, Xiaoping Cui, Xiaoyang Wang

**Affiliations:** 1 Fuzong Teaching Hospital, Fujian University of Traditional Chinese Medicine (900th Hospital), Fuzhou, China; 2 College of Integrative Medicine, Fujian University of Traditional Chinese Medicine, Fuzhou, China

**Keywords:** aging, cortical dedifferentiation, disease epicenter, morphometric inverse divergence, network neuroscience, structural connectivity, type 2 diabetes mellitus

## Abstract

**Background:**

Type 2 diabetes mellitus (T2DM) is associated with widespread cortical alterations and cognitive decline. However, the macroscopic organizational principles of T2DM-associated cortical degradation remain poorly understood. This study investigated whether T2DM-related cortical morphological dedifferentiation follows specific topological network principles.

**Methods:**

We analyzed structural MRI data from 49 T2DM patients and 49 matched healthy controls (HCs), representing an aging-relevant middle-aged and older-adult cohort. Individualized cortical morphometric similarity networks were constructed using the morphometric inverse divergence (MIND) method. Integrating these networks with normative functional and structural connectomes, we tested classic hub vulnerability, mapped candidate disease-specific epicenters, and evaluated structure-function alignment.

**Results:**

T2DM patients exhibited cortical dedifferentiation, particularly within the primary visual and dorsal attention networks. Spatial permutation testing revealed this distribution did not conform to generic hub vulnerability or uniform local spreading models. Instead, it preferentially aligned with cross-modal candidate epicenters, primarily in the superior parietal, superior frontal, and lingual regions. Crucially, patient-tailored modeling demonstrated a distinct structure-function divergence: individualized dedifferentiation maps correlated significantly with structural, but not functional, epicenter connectivity profiles. Additionally, regional disease duration effects correlated negatively with the structural epicenter profile, tentatively reflecting a ceiling effect in core sensory regions.

**Conclusion:**

The group-level spatial map showed exploratory correspondence with selected normative functional connectivity (FC)- and structural connectivity (SC)-based candidate profiles. Individualized profiles showed greater cross- sectional correspondence with the selected SC-based profile than with the FC-based profile, providing a network-informed description of diabetic brain alterations.

## 1 Introduction

Type 2 diabetes mellitus (T2DM) is a systemic metabolic disorder with increasing relevance to aging neuroscience because it commonly affects middle-aged and older adults and is associated with an elevated risk of cognitive decline, dementia and brain structural abnormalities (Moran et al., 2019; Roy et al., 2023; Zhang et al., 2022). High-resolution magnetic resonance imaging (MRI) has revealed widespread cortical atrophy and white matter microstructural degradation in diabetic patients (Gao et al., 2024; Li et al., 2020). These diabetes-related alterations may occur in the context of age-related changes in cortical morphology and white-matter integrity, making it important to distinguish metabolic and vascular effects from broader aging-related vulnerability. However, conventional neuroimaging studies have predominantly relied on isolated single-morphological metrics, such as cortical thickness or gray matter volume, to map independent regional deficits (Ajilore et al., 2010; Hansen et al., 2022; Roy et al., 2020).

Yet the field has largely interpreted these findings through a regional deficit framework, asking which cortical territories are affected rather than whether the organizational principles of the cortex itself are altered. This limitation is non-trivial. The cerebral cortex is a hierarchically differentiated system in which sensory, attentional, and transmodal territories preserve distinct morphometric identities that support increasingly integrative forms of cognition (Taylor et al., 2026; Wei et al., 2024). From this perspective, T2DM-related brain changes may not simply reflect scattered atrophic lesions; they may also indicate disruption of cortical differentiation at the macroscale. Reframing diabetic brain injury in this way shifts the emphasis from focal abnormality to disturbed cortical organization. This broader view brings cortical morphological homogenization into focus. Morphological homogenization, or cortical dedifferentiation, refers to a reduction in the normal morphometric distinctiveness of cortical regions, such that areas that are ordinarily differentiated become more structurally similar (García-San-Martín et al., 2025; Li et al., 2025). Conceptually, this is important because it captures coordinated remodeling of cortical architecture rather than isolated reductions in volume or thickness. Such a framework may be especially relevant to T2DM, where chronic metabolic stress and multidomain cognitive dysfunction together imply a distributed systems-level burden rather than a single-region pathology (Li et al., 2024; Zhang et al., 2025).

To bridge this gap, the morphometric inverse divergence (MIND) method offers a compelling and sensitive methodological framework (Sebenius et al., 2023). Unlike conventional group-level structural covariance (Seidlitz et al., 2018; Sun et al., 2023), MIND integrates multiple macroscopic morphological features to estimate the inter-regional morphometric similarity for each single patient, which may partly reflect shared cytoarchitectural organization.

**FIGURE 1:**
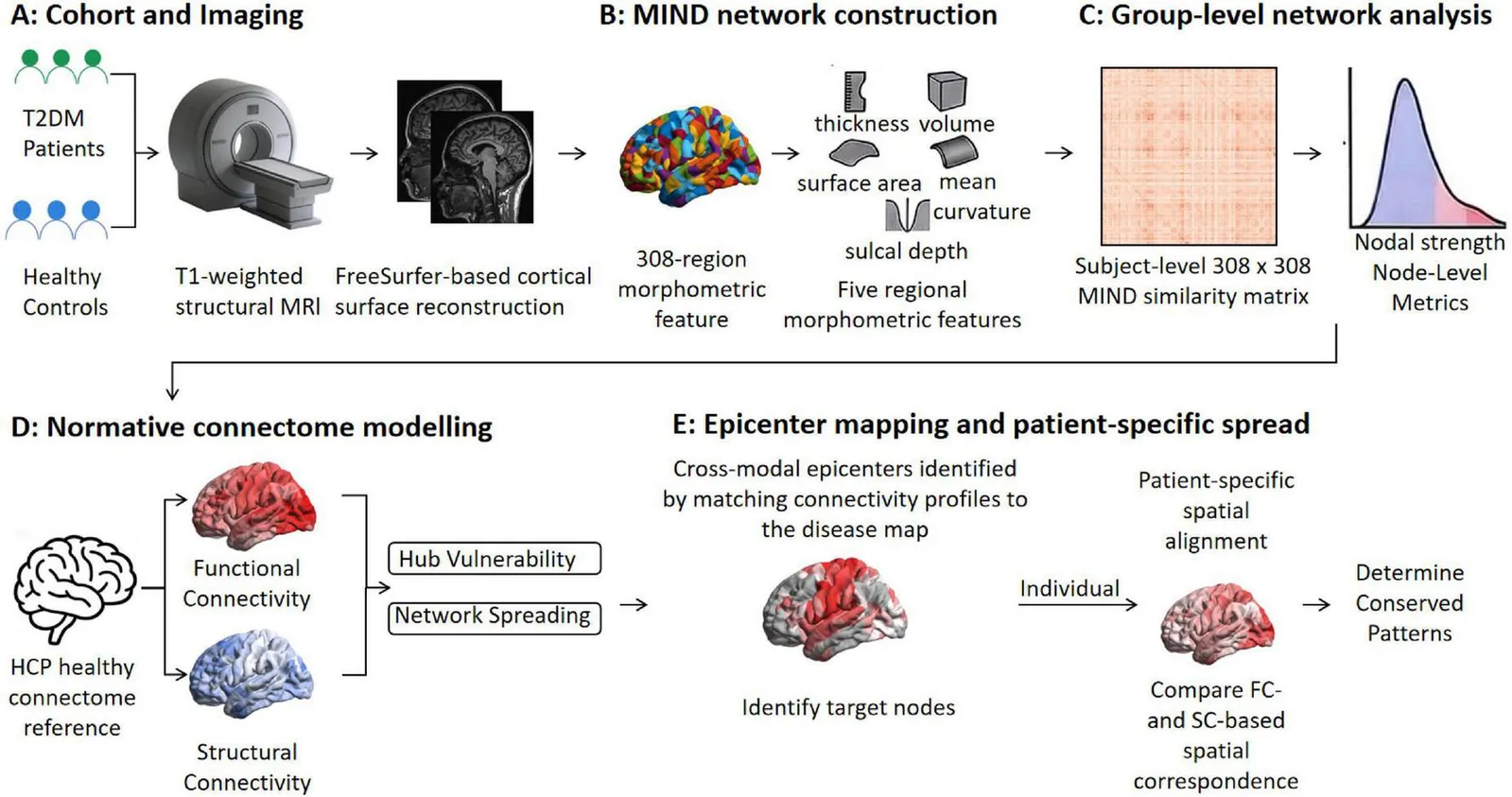
Schematic overview of the analytical framework. **(A)** Cohort and imaging preprocessing: High-resolution T1-weighted structural MRI data were acquired from patients with T2DM and HCs. Cortical surfaces were reconstructed and parcellated into 308 regions. **(B)** Construction of individualized MIND networks: Five macroscopic morphometric features (cortical thickness, volume, surface area, mean curvature, and sulcal depth) were extracted for each region. The MIND was computed to generate a subject-specific morphometric similarity matrix. **(C)** Group-level morphological profiling: Regional morphological dedifferentiation (nodal strength) was calculated. Group differences were evaluated using GLMs to identify significant cortical dedifferentiation at both regional and Yeo 7-network levels. **(D)** Normative connectome modeling and epicenter mapping: Normative functional FC and SC reference networks were derived from the HCP. We systematically tested the generic hub vulnerability model and mapped cross-modal disease epicenters by correlating normative connectivity profiles with the T2DM-associated dedifferentiation map. **(E)** Patient-tailored propagation and spatiotemporal dynamics: Individualized dedifferentiation maps were generated to evaluate patient-specific propagation along FC and SC epicenter networks, directly testing for structure-function dissociation. Finally, spatial associations with disease duration and cross-subject conserved epicenters were assessed. T2DM, type 2 diabetes mellitus; HCs, healthy controls; MRI, magnetic resonance imaging; MIND, morphometric inverse divergence; GLM, general linear model; FC, functional connectivity; SC, structural connectivity; HCP, Human Connectome Project.

Therefore, integrating individualized MIND profiling with normative functional and structural connectomes provides a valuable framework to investigate not only how the cortical architecture is reorganized, but also whether the spatial distribution of these alterations is aligned with normative topological organization. In the present study, we aimed to elucidate the macroscopic organizational principles underlying cortical morphological dedifferentiation in patients with T2DM. By integrating patient-specific MIND networks with normative functional and structural connectomes, we tested whether the cross-sectional spatial distribution of T2DM-associated MIND differences corresponded to generic hubness, connectome-weighted neighborhood structure or selected normative connectivity profiles. Furthermore, we evaluated whether these candidate-profile associations were evident in individualized maps and in spatial maps of disease-duration effects. We hypothesized that T2DM-associated MIND differences would show spatial correspondence with selected normative structural-connectivity profiles, without presupposing a temporal direction of change. An overview of the analytical framework is summarized in Figure 1.

## 2 Materials and methods

### 2.1 Participants and clinical evaluation

The MRI and clinical data were originally collected between May 2014 and December 2015. The present retrospective secondary analysis was approved by the Biomedical Ethics Committee under Approval No. 2026-043. Demographically matched healthy controls (HCs) were concurrently recruited from the hospital’s health examination center. The cohort represented an aging-relevant adult sample rather than an exclusively geriatric cohort. The inclusion criteria for the T2DM group required a definitive diagnosis according to the 1999 World Health Organization criteria (Alberti and Zimmet, 1998), as confirmed by senior endocrinologists. For the HC group, participants were required to have no history of glucose intolerance or family history of diabetes, with fasting blood glucose (FBG) below the diagnostic threshold. All participants were right-handed.

Potential participants were excluded if they presented with severe diabetic complications (e.g., stage 3 or higher diabetic nephropathy, recent diabetic ketoacidosis), a history of traumatic brain injury, stroke, epilepsy, severe substance abuse, organic brain lesions, or other psychiatric disorders. Potential participants were excluded according to the prespecified clinical and neurological criteria. The final cohort comprised 49 participants with T2DM and 49 HCs; all 98 participants subsequently passed the predefined imaging quality-control review.

### 2.2 Neuroimaging data acquisition and preprocessing

Clinical and neuropsychological evaluations were conducted prior to MRI scanning, starting with venous blood collection after an overnight fast ( > 8 h) to measure FBG levels using an automated biochemical analyzer. During the baseline period, global cognitive function was quantified by a trained neuropsychological evaluator blinded to the participants’ clinical status, using the Mini-Mental State Examination (MMSE) to provide standardized scoring.

Neuroimaging data were acquired using a 3.0T Siemens Magnetom Trio Tim scanner. High-resolution 3D T1-weighted structural images were obtained via an MP-RAGE sequence (TR = 1900 ms, TE = 2.52 ms, flip angle = 9°, matrix = 256 × 256, FOV = 250 × 250 mm^2^, slice thickness = 1.0 mm, no interslice gap, 176 contiguous sagittal slices). Subsequently, images were preprocessed for cortical surface reconstruction using FreeSurfer (v8.1.0)^1^ (Fischl, 2012), which included skull stripping, tissue segmentation, surface reconstruction, and spherical normalization. The reconstructed pial and white matter surfaces of all participants were visually inspected to ensure topological accuracy.

A total of 98 participants were included in the final analysis, comprising 49 participants with T2DM and 49 HCs. To leverage modern computational advancements, the raw 3D T1-weighted structural images, originally acquired and archived between 2014 and 2015, were recently processed from scratch using the modern FreeSurfer (v8.1.0) pipeline for this specific retrospective analysis. The reconstructed pial and white-matter surfaces were visually inspected for skull-stripping errors, inaccurate gray–white or pial boundaries, topological defects, and major segmentation failures. All 98 participants passed the predefined visual quality-control criteria, and no participant was excluded after quality-control review. Crucially, to prevent potential observer bias, the quality-control assessor was strictly blinded to the clinical group allocations. Prior to visual inspection, all structural MRI data were pseudonymized, and participant IDs were replaced with randomized alphanumeric codes, ensuring that the assessor had no access to demographic or diagnostic information during the quality-control process. Furthermore, no manual topological edits were required for the included participants.

### 2.3 Construction of MIND similarity matrix

The Desikan-Killiany atlas was further segmented into 308 spatially adjacent cortical areas (Desikan et al., 2006; Seidlitz et al., 2018). Cortical morphometric similarity networks were constructed using the Python implementation of morphometric inverse divergence (MIND). For each participant, five vertex-wise FreeSurfer features were used: surface area, mean curvature, cortical thickness, volume and sulcal depth. The features were mapped to the 308-region cortical parcellation. Within each participant, each feature was standardized across all retained cortical vertices by subtracting its whole-cortex mean and dividing by its whole-cortex standard deviation. The current implementation did not apply the optional vertex-exclusion or outlier-removal procedures. For every pair of cortical regions, the multivariate feature distributions were compared using a symmetric Kullback-Leibler (KL) divergence estimated from nearest-neighbor distances (see Equation 1).


$$M\,I\,N\,D\,\left(a,b\right)=\frac{1}{1+K\,L\,\left(a,b\right)}$$ (1)

These values were transformed into a similarity measure ranging from 0 to 1, constructing a 308 × 308 MIND similarity matrix for each subject.

In this study, “cortical dedifferentiation” is used as an operational term for increased regional nodal strength in the MIND similarity network. This measure indicates that the multivariate morphometric profile of a region is more similar to the profiles of other cortical regions within the MIND representation. It should not be interpreted as a direct measurement of cytoarchitectural convergence, neuronal identity or a causal biological process. Increased nodal strength may also be influenced by global morphometric effects, the relative contribution of individual features and the feature-standardization procedure.

### 2.4 Regional and network-level morphological profiling

Following the standard ENIGMA^2^ (Larivière et al., 2021) workflow, dimensionality reduction was performed by calculating the nodal strength for each cortical region (i.e., its average connection weight with all other regions). To identify disease-related morphological alterations, node-wise general linear models (GLMs) were constructed to compare the nodal strength profiles between patients and HCs, rigorously controlling for age, sex, and the age-by-sex interaction term. A positive t-statistic (*t* > 0) indicated an increased morphological dedifferentiation (i.e., dedifferentiation) in patients. Findings across the 308 areas were corrected using the false discovery rate (FDR) procedure (Benjamini and Hochberg, 1995). Furthermore, regions were mapped to the Yeo 7 networks (Yeo et al., 2011). Group comparisons for these macroscale networks were performed using the identical GLM design and FDR correction.

### 2.5 HCP normative network generation

We utilized structural and functional imaging data from healthy young adults (*n* = 207; age range: 22–35 years) provided by the Human Connectome Project (HCP). These matrices were normative reference maps and were not measured in the T2DM participants. The detailed recruitment procedures, standardized neuroimaging acquisition parameters (e.g., spatial resolution and scanner protocols), and comprehensive preprocessing pipelines for this normative cohort have been extensively documented in previous literature (Van Essen et al., 2012). Normative functional connectivity (FC) matrices were generated by computing pairwise correlations between resting-state fMRI time series. Normative structural connectivity (SC) matrices were generated using anatomically-constrained tractography via MRtrix3 (Tournier et al., 2019), applying SIFT2 (Smith et al., 2015) to reconstruct whole-brain streamlines.

To examine whether the spatial distribution of T2DM-associated MIND differences corresponds to the intrinsic organization of a disease-independent human connectome, we used normative functional and structural connectivity matrices derived from the Human Connectome Project. These matrices were used as reference architectures and were not interpreted as measurements of the patients’ own connectivity. This design addresses a different question from patient-specific connectomics: it tests whether the group-level morphological difference pattern is spatially organized according to a normative network scaffold. In contrast, patient-specific functional or structural connectivity would quantify disease-related connectivity abnormalities, but could also incorporate disease-related network reorganization, transient cognitive or metabolic state effects, and imaging-quality variation. Accordingly, normative connectivity was used as an independent reference framework for spatial correspondence analyses, rather than as a proxy for the patients’ actual anatomical or functional pathways.

### 2.6 Connectome architecture and disease epicenter mapping

To investigate whether T2DM-induced morphological dedifferentiation was shaped by the normative connectome, we established the global nodal stress model and the targeted epicenter model. First, we performed spatial correlation analyses between the morphological dedifferentiation profiles (GLM-derived t-values) and normative FC/SC degree centrality maps. Second, we mapped disease epicenters by systematically correlating the normative connectivity profile of every cortical region with the whole-brain dedifferentiation map. Regions exhibiting significant spatial similarity and positive correlation (*r* > 0) were ranked, with the top-ranked regions identified as candidate primary epicenters. For epicenter discovery across the 308 seeds, we evaluated statistical significance using spatial permutation tests (P_*spin*_ < 0.05) and selected the top five highest-ranked epicenters.

### 2.7 Association with disease duration

To examine whether the cross-sectional spatial pattern of disease-duration effects was associated with normative connectome organization, we performed a within-patient analysis to extract the spatial pattern of disease duration effects. Node-wise GLMs were applied exclusively within the T2DM group, defining regional nodal strength as the dependent variable, disease duration as the independent variable, and strictly adjusting for age, sex, and the age-by-sex interaction. The resulting t-statistic map of disease duration was subsequently spatially correlated with both the normative degree centrality maps (hubness) and the specific connectivity profiles of the exploratory candidate regions. Because disease duration was retrospectively recorded and imaging was acquired at a single time point, this analysis was interpreted as a spatial association and not as a longitudinal progression analysis.

### 2.8 Spatial permutation tests (spin test)

To account for the intrinsic spatial autocorrelation of cortical maps (Alexander-Bloch et al., 2018), the statistical significance of all spatial associations (including nodal stress, epicenter mapping, and duration associations) was rigorously assessed using spatial permutation tests. Null models were generated by projecting the spatial coordinates of the 308 cortical parcel centroids onto a surface sphere, applying randomly sampled rotations (10,000 repetitions), and reassigning the empirical values to the nearest rotated parcels to calculate the P_*spin*_.

### 2.9 Patient-tailored epicenter network alignment

To characterize inter-individual heterogeneity, regional nodal strength for each T2DM participant was standardized relative to the HC reference distribution to generate individualized morphological profiles. These profiles were then spatially correlated with normative FC- and SC-based connectivity profiles defined from the group-level candidate epicenter analysis. Right-tailed Wilcoxon signed-rank tests were used to assess whether the patient-specific correlations were shifted above zero, and a paired Wilcoxon signed-rank test compared the FC- and SC-based correlations. Patient-specific epicenter maps were considered exploratory and were interpreted with the same caution regarding multiple testing and sample dependence.

The primary group-level analysis was treated as an internal consistency analysis because the candidate profiles and individualized maps were derived from the same cohort. As a sensitivity analysis, we performed leave-one-T2DM-participant-out validation. For each held-out participant, the group-level map and candidate FC/SC profiles were recomputed using the remaining 48 T2DM participants and the HC reference group; the held-out participant’s individualized map was then correlated with the resulting profiles. This procedure reduced dependence between patient-level evaluation and candidate-seed selection but did not remove all dependence associated with the common HC reference.

### 2.10 Statistical analysis of clinical data

Demographic and clinical characteristics were analyzed using MATLAB software (R2024b). The normality of continuous variables was assessed using the Shapiro-Wilk test. Normally distributed continuous variables were expressed as mean ± standard deviation and compared between groups using independent two-sample *t*-tests. Non-normally distributed continuous variables were presented as median and interquartile range (Q1, Q3) and compared using the non-parametric Mann-Whitney U test. Categorical variables were expressed as counts and compared between groups using the Chi-square (χ^2^) test. A two-tailed *P* < 0.05 was considered statistically significant.

## 3 Results

### 3.1 Demographics

There were no significant differences between the T2DM and HC groups in terms of age (*Z* = 1.818, *P* = 0.069), sex (χ^2^ = 0.367, *P* = 0.544), or years of education (*Z* = −0.740, *P* = 0.460), indicating that the two groups were well-matched demographically. However, patients in the T2DM group exhibited significantly higher FBG levels compared to the HC group (*Z* = 8.150, *P* < 0.001). Furthermore, the T2DM group demonstrated relative cognitive decline, as evidenced by significantly lower scores on the MMSE (*Z* = −4.192, *P* < 0.001). The detailed demographic and clinical characteristics of the participants are summarized in Table 1.

**TABLE 1:** Demographic and clinical characteristics of the study participants.

Characteristics	T2DM group	HC group	Z/χ^2^ value	*P*-value
Age (years)	59.00 (53.00, 65.25)	58.00 (50.75, 60.00)	1.818^a^	0.069
Sex (male/female)	26, 23	23, 26	0.367^b^	0.544
Education (years)	7.00 (6.00, 11.00)	8.00 (6.00, 11.00)	−0.740^a^	0.460
Disease duration (years)	10.30 ± 5.97	N/A	–	–
FBG (mmol/L)	8.60 (7.35, 11.84)	5.33 (4.70, 5.73)	8.150^a^	< 0.001
MMSE (score)	27.00 (24.00, 29.00)	29.00 (27.75, 30.00)	−4.192^a^	< 0.001

Data are presented as median (Q1, Q3) for continuous variables and as counts for categorical variables. T2DM, type 2 diabetes mellitus; HC, healthy control; FBG, fasting blood glucose; MMSE, Mini-Mental State Examination.

^a^*Z*-values derived from the Mann-Whitney U test;

^b^χ^2^ value derived from the Chi-square test.

### 3.2 Cortical morphological dedifferentiation patterns in T2DM

**FIGURE 2:**
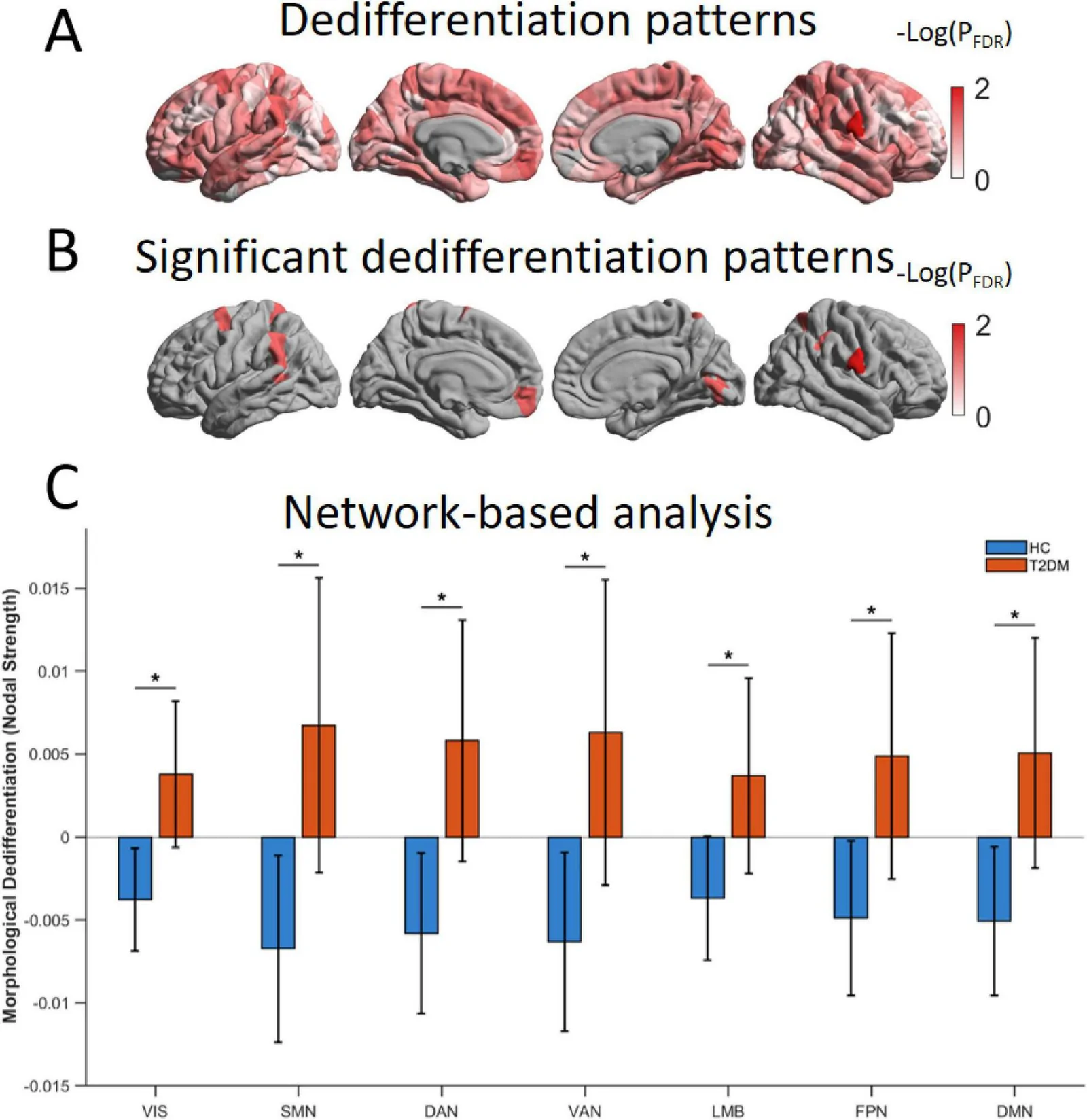
Cortical morphological dedifferentiation and macroscopic network susceptibility in T2DM. **(A)** Unthresholded spatial distribution of group-level morphological dedifferentiation differences between T2DM patients and HCs. The statistical maps are projected onto the 308-region cortical surface. The color bar indicates the -log(P_FDR_) values, reflecting the statistical magnitude of increased nodal strength (i.e., dedifferentiation) in patients. **(B)** Cortical regions exhibiting statistically significant morphological dedifferentiation after correcting for multiple comparisons using the strict FDR procedure (P_FDR_ < 0.05). **(C)** Network-based analysis mapping the regional dedifferentiation profiles to the seven canonical Yeo resting-state FC networks. The grouped bar chart illustrates the mean nodal strength for each macroscale network in the HC and T2DM groups. Error bars represent 95% confidence intervals. Asterisks denote statistically significant group differences (P_FDR_ < 0.05).*T2DM, type 2 diabetes mellitus; HCs, healthy controls; FDR, false discovery rate; VIS, visual network; SMN, somatomotor network; DAN, dorsal attention network; VAN, ventral attention network; LMB, limbic network; FPN, frontoparietal network; DMN, default mode network; GLM, general linear model; FC, functional connectivity.

Our node-wise GLM analysis identified 13 cortical parcels with increased MIND nodal strength in the T2DM group after FDR correction (P_*FDR*_ < 0.05; Figures 2A, B and Supplementary Table 1). The significant parcels included the left banks of the superior temporal sulcus, left caudal middle frontal, left medial orbitofrontal, left superior frontal, left superior parietal and three left supramarginal parcels, as well as right lingual, right pericalcarine, right postcentral, right superior parietal and right supramarginal parcels. The largest effect was observed in the right postcentral parcel (part4, *t* = 4.646, P_*FDR*_ = 0.0034). Parcel-level labels and statistics are reported exactly as listed in Supplementary Table 1.

Furthermore, to characterize these topological reconfigurations at a macroscopic scale, we aggregated the regional dedifferentiation profiles into the seven canonical resting-state FC networks (Yeo 7 networks). Although only 13 parcels survived the stringent region-wise FDR correction, network-level averaging captured distributed subthreshold shifts, revealing that morphological dedifferentiation in T2DM is a widespread, system-level phenomenon. All seven macroscale networks demonstrated significantly elevated dedifferentiation compared to HCs (all P_*FDR*_ < 0.05, Figure 2C). Among them, the primary visual network (VIS; *t* = 2.887, P_*FDR*_ = 0.023) and the dorsal attention network (DAN; *t* = 2.730, P_*FDR*_ = 0.023) exhibited the most pronounced dedifferentiation. This was closely followed by profound alterations in the somatomotor (SMN; *t* = 2.631, P_*FDR*_ = 0.023), default mode (DMN; *t* = 2.514, P_*FDR*_ = 0.024), ventral attention (VAN; *t* = 2.427, P_*FDR*_ = 0.024), frontoparietal (FPN; *t* = 2.285, P_*FDR*_ = 0.029), and limbic (LMB; *t* = 2.165, P_*FDR*_ = 0.033) networks. These network-level findings corroborate our regional analyses, highlighting that while T2DM imposes a global dedifferentiation burden on the cortical connectome, primary sensory and attentional networks are particularly vulnerable.

### 3.3 Evaluation of global hub vulnerability and network spreading

**FIGURE 3:**
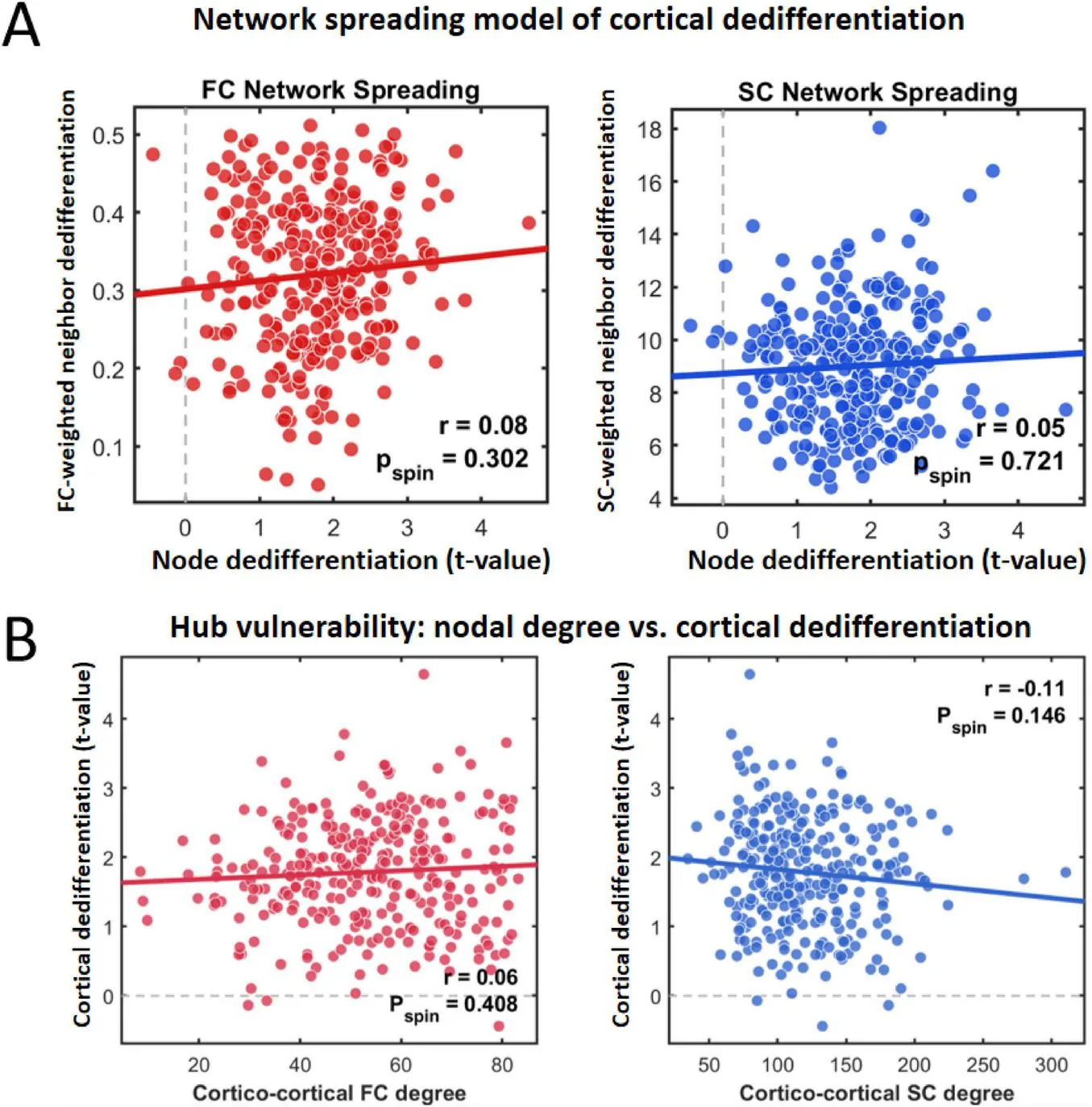
Spatial relationships between regional cortical dedifferentiation and normative connectome architecture. **(A)** Network spreading model. Scatter plots display the spatial correlations between the intrinsic node dedifferentiation (GLM-derived *t*-values, *x*-axis) and the connectome-weighted mean neighbor dedifferentiation (*y*-axis) based on the FC (red) and SC (blue) networks. **(B)** Hub vulnerability model. Scatter plots illustrate the spatial correlations between normative cortico-cortical nodal degree (*x*-axis) and regional cortical dedifferentiation (*y*-axis) across the FC (red) and SC (blue) networks. In both panels, the solid lines represent the linear fit. The empirical correlation coefficients (r) and the corresponding spatial permutation *p*-values (P_spin_, 10,000 repetitions) are annotated within each plot. Each point represents one of the 308 cortical parcels, rather than an individual participant. GLM, general linear model; FC, functional connectivity; SC, structural connectivity.

We evaluated the spatial relationships between regional morphological dedifferentiation and normative network architecture. First, we tested the network spreading model by correlating the intrinsic dedifferentiation of each cortical node (GLM-derived t-values) with the connectome-weighted mean dedifferentiation of its topological neighbors (Figure 3A). Assessed via spatial permutation testing, our analysis revealed no significant correlations for either the FC (*r* = 0.08, P_*spin*_ = 0.302) or SC (*r* = 0.05, P_*spin*_ = 0.721) networks.

Next, we investigated the global hub vulnerability model by assessing the spatial correlations between the regional dedifferentiation profiles and normative cortico-cortical nodal degree (Figure 3B). Similarly, we observed no significant spatial associations across the FC (*r* = 0.06, P_*spin*_ = 0.408) and SC (*r* = −0.11, P_*spin*_ = 0.146) connectomes.

### 3.4 FC and SC disease epicenters

**FIGURE 4:**
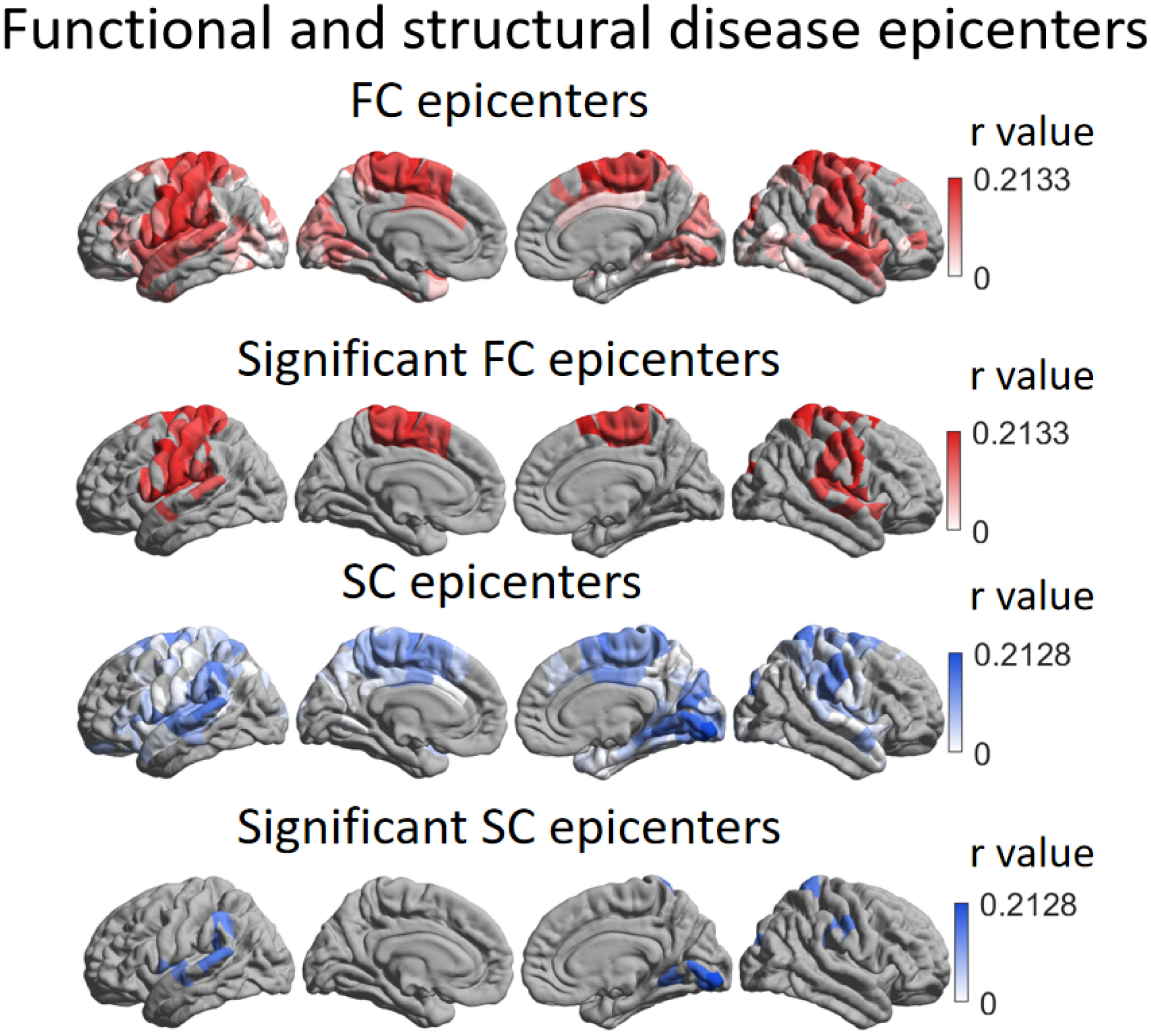
FC and SC disease epicenters of cortical morphological dedifferentiation. Disease-specific epicenters were identified by systematically correlating the normative FC and SC profiles of each cortical region with the group-level morphological dedifferentiation map. The top two rows display the unthresholded FC epicenter map and the statistically significant FC epicenters (assessed via 10,000-repetition spatial spin tests, P_spin_ < 0.05, red color scale). The bottom two rows display the corresponding unthresholded SC epicenter map and the statistically significant SC epicenters (P_spin_ < 0.05, blue color scale). The color bars indicate the empirical Pearson correlation coefficients (r), representing the spatial similarity between a region’s normative connectivity network and the actual distribution of T2DM cortical dedifferentiation. FC, functional connectivity; SC, structural connectivity; T2DM, type 2 diabetes mellitus.

Given the divergence from generic global hub vulnerability, we systematically mapped disease-specific epicenters to identify cortical regions whose normative connectivity profiles spatially resembled the T2DM-induced morphological dedifferentiation map (Figure 4).

In the FC connectome, spatial permutation testing identified a specific set of significant disease epicenters (P_*spin*_ < 0.05, *r* > 0). The primary FC epicenters, exhibiting the highest spatial correlations, were predominantly localized to the right superior parietal lobule (peak *r* = 0.21, P_*spin*_ = 0.006), right superior frontal gyrus, and bilateral precentral gyri. Similarly, in the SC connectome, the primary epicenters governing the spatial distribution of pathology were highly concentrated in the right lingual gyrus (peak *r* = 0.21, P_*spin*_ = 0.001), right superior parietal lobule, and right supramarginal gyrus.

Notably, these highly ranked cross-modal epicenters (e.g., the right superior parietal lobule and right lingual gyrus) spatially converged with the core cortical regions exhibiting the most profound morphological dedifferentiation identified in our regional analysis. The complete statistical details, including the correlation coefficients and P_*spin*_ for all 308 cortical regions across both FC and SC networks, are provided in Supplementary Table 2.

### 3.5 Spatial associations between disease duration and connectome organization

We performed a within-patient analysis to extract the spatial pattern of disease duration effects. The resulting t-statistic map of disease duration was subsequently spatially correlated with both the normative degree centrality maps (hubness) and the specific connectivity profiles of the exploratory candidate regions.

**FIGURE 5:**
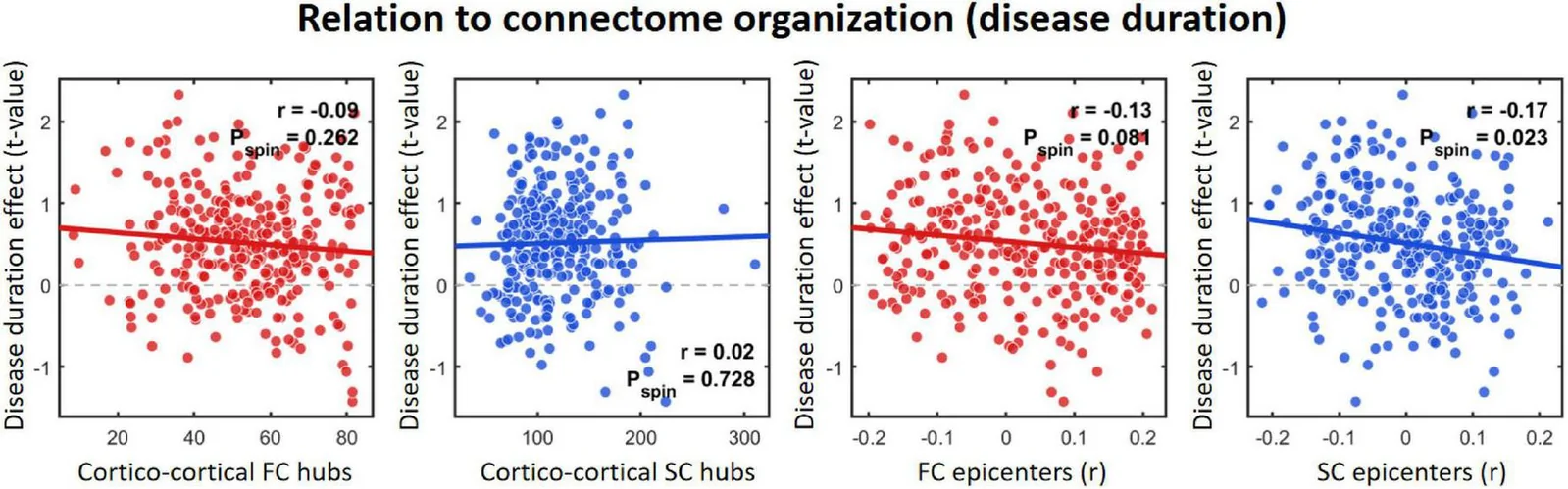
Spatial associations between the disease duration effect and connectome organization. Scatter plots illustrate the spatial correlations between the regional disease duration effect (within-patient GLM-derived t-values, *y*-axis) and four distinct network properties (*x*-axis): cortico-cortical FC hubs, SC hubs, FC epicenter connectivity profile, and SC epicenter connectivity profile. The solid lines represent the linear fit. The empirical Pearson correlation coefficients (r) and the corresponding spatial permutation *p*-values (P_spin_, 10,000 repetitions) are annotated within each panel. Each point represents one of the 308 cortical parcels, rather than an individual participant. GLM, general linear model; FC, functional connectivity; SC, structural connectivity.

As illustrated in Figure 5, spatial permutation testing revealed no significant spatial correlations between the regional disease duration effect and normative FC (*r* = −0.09, P_*spin*_ = 0.262) or SC (*r* = 0.02, P_*spin*_ = 0.728) nodal degree. Similarly, the spatial association with the FC epicenter connectivity profile did not reach statistical significance (*r* = −0.13, P_*spin*_ = 0.081). A weak negative spatial association was observed between the disease-duration effect map and the normative SC-based epicenter profile (*r* = −0.17, P_*spin*_ = 0.023). This association was modest and should be interpreted as a cross-sectional spatial pattern rather than evidence of temporal progression.

### 3.6 Patient-tailored epicenter network alignment and conserved regions

**FIGURE 6:**
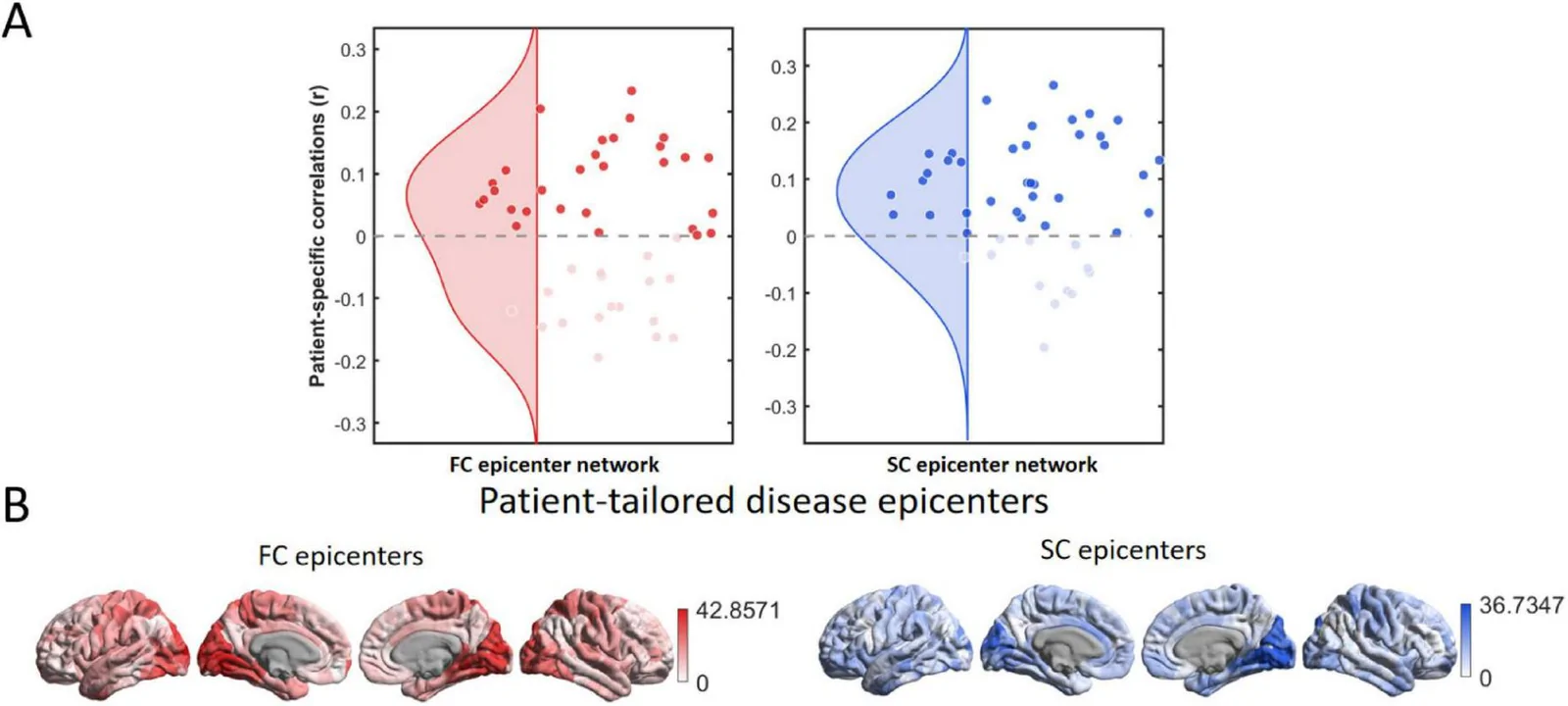
Patient-tailored spatial correspondence with candidate connectivity profiles and exploratory individual-region hit rates. **(A)** Raincloud plots illustrate the distribution of patient-specific spatial correlations (r, *y*-axis) between individualized morphological dedifferentiation maps and the normative connectivity profiles seeded from the primary group-level epicenters. The FC epicenter network is depicted in red, and the SC epicenter network in blue. Each scatter point represents an individual T2DM patient. A right-tailed Wilcoxon signed-rank test was utilized to evaluate whether the cohort exhibited statistically significant positive spatial correspondence. **(B)** Unthresholded probability maps displaying the cross-subject conservation probability of individual epicenters. Patient-specific epicenters were identified via 1,000-repetition spatial permutation tests. The color bars indicate the percentage of patients (%) in whom a given cortical region met the exploratory spatial-permutation threshold for an FC (red, left)- or SC (blue, right)-based candidate profile. T2DM, type 2 diabetes mellitus; FC, functional connectivity; SC, structural connectivity.

To characterize inter-individual heterogeneity, we examined whether individualized morphological profiles showed spatial correspondence with the normative connectivity profiles of the group-level candidate epicenters. We spatially correlated each patient’s individualized morphological dedifferentiation map with the normative connectivity profiles seeded from the selected group-level FC- and SC-based candidate regions (Figure 6A). Evaluated via the right-tailed Wilcoxon signed-rank test, the spatial correlations within the FC epicenter network did not significantly deviate from zero (median *r* = 0.037, *P* = 0.122, effect size *r* = 0.19), with 63.27% of patients exhibiting positive spatial correlations. In contrast, individualized dedifferentiation patterns showed a statistically significant positive spatial correlation with the SC epicenter network (median *r* = 0.070, *P* < 0.001, effect size *r* = 0.63), with 73.47% of T2DM patients showing positive spatial correlations with the normative SC-based profile. This result indicates cross-sectional spatial correspondence and should not be interpreted as evidence of longitudinal propagation. Direct comparison revealed that the patient-specific alignment with the SC epicenter network was significantly stronger than that with the FC epicenter network (*Z* = 2.054, *P* = 0.040, effect size *r* = 0.34).

Furthermore, we localized idiosyncratic epicenters for each patient using 1,000-repetition spatial permutation tests to calculate the cross-subject conservation probability (Figure 6B). Despite immense individual clinical variance, the highest-probability patient-specific epicenters were recurrent in a subset of participants and predominantly converged on the primary visual and occipital cortices.

Crucially, these repeatedly observed candidate regions showed overlap between the FC- and SC-based exploratory maps. For instance, the right pericalcarine cortex and bilateral lingual gyri emerged as highly conserved idiosyncratic epicenters across both FC (up to 38.78% of the cohort) and SC (up to 36.73% of the cohort) connectomes. The comprehensive cross-subject conservation probabilities for all 308 cortical regions are detailed in Supplementary Table 3.

Because the group-level candidate epicenters and the individualized profiles were originally derived from the same cohort, we further implemented a rigorous leave-one-out cross-validation (LOOCV) framework as a sensitivity analysis to mitigate potential analytical circularity and selection bias. Detailed methodology and results of this held-out sensitivity analysis are provided in Supplementary Methods. This sensitivity analysis yielded results highly consistent with our primary findings, confirming the robustness of the observed spatial correspondences.

## 4 Discussion

In this aging-relevant adult cohort, we investigated the macroscopic organizational principles underlying cortical morphological dedifferentiation in patients with T2DM. By integrating high-resolution morphometric similarity networks with normative connectome architecture, our findings suggest that T2DM-associated cortical dedifferentiation is a highly targeted, network-constrained process rather than simply a random global failure. Notably, we observed a potential divergence from classic hub-centric vulnerability models. Instead, the cross-sectional spatial pattern showed its strongest correspondence with several candidate cross-modal regions, predominantly in the visual and frontal cortices, and may preferentially propagate along the structural, rather than functional, connectome scaffold.

### 4.1 Cortical morphological dedifferentiation and epicenter mapping

Our regional and network-level profiling revealed a widespread, system-level morphological dedifferentiation across the T2DM cortex. In our spatial modeling, specific sensory and associative cortices, predominantly the superior parietal, superior frontal, and visual cortices (e.g., lingual gyrus), emerged as candidate cross-modal epicenters. At the macroscale network level, while all seven canonical Yeo networks exhibited elevated dedifferentiation, the primary VIS and DAN appeared to be the most profoundly affected. To reconcile why the regions showing the strongest cross-sectional association (epicenters) simultaneously display the most severe cumulative burden, our within-patient duration analysis offers a crucial mechanistic clue. We observed a statistically significant negative correlation between regional disease duration effects and the structural epicenter connectivity profile. By analogy with staged structural burden in other brain disorders (Denning et al., 2024; Mu et al., 2024), this negative association is indicative of a ceiling-like effect. The prominence of visual and related associative regions in the present cross-sectional maps is compatible with, but does not establish, their preferential vulnerability in T2DM. This premise is supported clinically, as retinal neurodegeneration, microvascular retinopathy, and subtle visual processing deficits are frequently observed early in T2DM (Liu et al., 2025; Zhang et al., 2023), sometimes occurring prior to clinically overt cognitive impairment (Crosby-Nwaobi et al., 2013; Hugenschmidt et al., 2014; Zhu et al., 2024). The weak negative association with disease duration may reflect a ceiling-like cross-sectional pattern, but longitudinal imaging is strictly required to determine whether these regions are affected earlier or reach a plateau over time.

### 4.2 Connectome architecture: hub vulnerability versus candidate-profile correspondence

A central hypothesis in network neuroscience posits that highly connected global hubs undergo the greatest pathological burden due to their topological centrality, a phenomenon often observed in primary neurodegenerative diseases (Larivière et al., 2020; Lin et al., 2025). However, our spatial permutation testing did not reveal a preferential vulnerability of functional or structural hubs in T2DM. Furthermore, the uniform connectome-weighted neighborhood model also did not significantly account for the spatial distribution of the pathology. This negative finding lends support to the view that T2DM cortical alterations might be better explained by selected FC- and SC-based candidate profiles that may reflect spatial heterogeneity in regional vulnerability, rather than solely by a generic topological failure characterized by indiscriminate network diffusion.

### 4.3 Divergence between structural and functional connectome alignment

Given this epicenter-driven spatial pattern, our multimodal modeling highlighted that normative SC profiles provided stronger explanatory alignment than normative FC profiles. Specifically, patient-tailored dedifferentiation patterns showed significant spatial alignment with the structural epicenter network, whereas functional epicenter alignment was non-significant. This structural alignment appears biologically plausible. T2DM-associated neurotoxicity, oxidative stress, and microangiopathy frequently manifest as cumulative white matter microstructural degradation (Alotaibi et al., 2025; Gao et al., 2024). Therefore, it is reasonable to hypothesize that normative SC profiles may provide a more stable reference for the observed spatial pattern for the spatial organization of T2DM-associated cortical dedifferentiation. Conversely, the weaker alignment with the functional connectome might be influenced by transient compensatory mechanisms. In response to early structural insults, the diabetic brain often engages in compensatory functional hyper-connectivity (Jing et al., 2023; Wang et al., 2022). This highly dynamic functional adaptation may mask a direct linear relationship with progressive morphological dedifferentiation, rendering functional connectivity a potentially weaker predictor of individual pathological spread in this context.

Taken together, patient-tailored analyses identified recurrent candidate epicenter-like regions in a subset of participants. Right pericalcarine and lingual parcels were among the repeatedly observed regions, with hit rates exceeding one-third of the cohort in selected FC- or SC-based exploratory maps. These findings indicate recurrence across individuals but do not establish universal conservation, highlighting the need for independent prospective validation.

### 4.4 Limitations

Several methodological limitations should be acknowledged. First, the cross-sectional design of this study precludes definitive causal inferences regarding the temporal sequence of spatial progression. Future longitudinal structural MRI studies are warranted to trace the dynamic spatial alignment of morphological dedifferentiation over time. Second, while our findings point toward microvascular and metabolic vulnerabilities, the exact molecular mechanisms, such as dynamic glycemic fluctuations or specific inflammatory markers, were not fully captured and require further investigation. Third, the connectome constraints were derived from normative architecture rather than patient-specific diffusion and functional MRI data, thus reflecting reference organizational scaffolds rather than individualized physical pathways. Fourth, the normative-connectome framework also has important limitations. The HCP cohort was substantially younger than the present clinical cohort, and differences in age-related network organization, metabolic and vascular status, scanner protocols, and preprocessing may influence the estimated spatial correspondence. Moreover, normative FC and SC cannot capture patient-specific white-matter injury or functional reorganization. Therefore, the present findings should be interpreted as correspondence with a healthy-population reference architecture, rather than evidence that the patients’ own structural or functional pathways caused or mediated the observed MIND pattern. Future studies should combine age-matched normative connectomes with patient-specific diffusion and functional MRI data. Fifth, several clinically relevant variables, including BMI, blood pressure, lipid-lowering and glucose-lowering medications, detailed vascular risk factors, and retinopathy status, were not consistently available in this retrospective dataset. HbA1c and complication-related variables were also incomplete for some participants. Therefore, the present analyses could not fully control for long-term glycemic exposure, treatment heterogeneity, vascular risk, or microvascular complications, meaning we cannot entirely exclude residual metabolic and vascular confounding. Future prospective studies should systematically incorporate these into preregistered sensitivity analyses. Sixth, the MIND index is a composite statistical measure rather than a direct assay of cytoarchitectural organization. Because the relative contribution of each feature was not independently estimated through single-feature or feature-removal sensitivity analyses, we cannot exclude the possibility that global atrophy, feature-specific variance, or the within-subject standardization procedure influenced nodal strength. Single-feature and feature-removal analyses should be performed in future work. Seventh, global cognition was solely evaluated using the MMSE, which lacks the sensitivity to detect subtle, domain-specific cognitive deficits such as executive, attention, or visuospatial dysfunctions frequently associated with T2DM. Therefore, the available clinical data cannot support domain-specific cognitive interpretations, and comprehensive neuropsychological batteries are required in future studies to establish the clinical validity of these network metrics. Finally, although our sample size was sufficient for robust spatial permutation testing, validation in larger, multi-center cohorts is necessary to assess the clinical generalizability of these patient-tailored connectomic models.

## 5 Conclusion

In conclusion, this study provides evidence that T2DM is associated with a specific, network-constrained pattern of cortical morphological dedifferentiation. Deviating from generic hub-centric models, the spatial organization of T2DM cortical pathology appears to be aligned with targeted, multi-modal candidate profiles involving visual and association regions, including lingual, superior parietal, and superior frontal parcels. Importantly, this epicenter-like spatial pattern exhibits stronger alignment with normative SC architecture than with functional networks. The spatial distribution further suggests a potential gradient-like cross-sectional organization from these heavily burdened sensory and associative regions. These findings provide an aging-relevant, patient-tailored connectomic perspective on diabetic brain alterations in middle-aged and older adults and offer cautious insights into the macroscopic organization of metabolic brain vulnerability.

## Data Availability

The original contributions presented in this study are included in this article/Supplementary material, further inquiries can be directed to the corresponding authors.

## Footnotes

1
https://surfer.nmr.mgh.harvard.edu/

2
https://enigma-toolbox.readthedocs.io/
