# Supplementary material for: Epicenter mapping of cortical morphological dedifferentiation in type 2 diabetes mellitus using morphometric inverse divergence
Source: Front Aging Neurosci. 2026 Aug 24;18:1884185. doi: 10.3389/fnagi.2026.1884185 (PMC13547275; doi:10.3389/fnagi.2026.1884185)
Supplement: Supplementary file 1 [file Table_4.docx]

**Supplementary Methods: Leave-one-out cross-validation of patient-tailored epicenter network alignment**

**Rationale:** In our main analysis, group-level candidate epicenters and individualized morphological profiles were derived from the same T2DM cohort. To address the potential risk of analytical circularity and selection bias, we conducted a rigorous independent validation using a leave-one-out cross-validation (LOOCV) framework.

**Methods:** For each of the 49 T2DM participants, the individual was iteratively excluded. A group-level cortical dedifferentiation map was then derived exclusively from the remaining 48 participants (the training set) to independently identify the candidate functional (FC) and structural (SC) epicenters. Subsequently, the individualized morphological profile of the single excluded participant (the test set) was spatially correlated with the normative connectivity profiles seeded from these independently derived epicenters.

**Results:** The primary candidate epicenters identified across the 49 independent training folds demonstrated remarkable anatomical stability. Specifically, the SC epicenters were uniformly anchored in the right lingual gyrus across all 49 folds, confirming the robustness of the core pathological nexus. Crucially, this strictly independent validation confirmed our primary findings. Evaluated via the right-tailed Wilcoxon signed-rank test, the spatial correlations within the independent FC epicenter networks did not significantly deviate from zero (median r = 0.008, P = 0.249), with 55.10% of patients exhibiting positive spatial correlations. In contrast, individualized dedifferentiation patterns showed a statistically significant positive spatial correlation with the SC epicenter network (median r = 0.058, P = 0.001), with 69.39% of T2DM patients showing positive alignment. Direct comparison re-confirmed that the patient-specific alignment with the SC epicenter network was significantly stronger than that with the FC epicenter network (Z = 2.054, P = 0.040, effect size r = 0.29). These LOOCV results demonstrate that our findings regarding structure-function divergence are highly robust and not driven by circular analysis.

**Fig. Patient-tailored epicenter network alignment and individual disease epicenter probability via LOOCV.** (A) Raincloud plots illustrate the distribution of strictly independent patient-specific spatial correlations (r, y-axis) between individualized morphological dedifferentiation maps and the normative connectivity profiles seeded from the candidate epicenters derived exclusively from the N-1 training sets. The FC epicenter network is depicted in red, and the SC epicenter network in blue. Each scatter point represents an individual T2DM patient. A right-tailed Wilcoxon signed-rank test was utilized to evaluate whether the cohort exhibited statistically significant positive spatial correspondence. (B) Unthresholded probability maps displaying the cross-subject conservation probability of individual epicenters. Patient-specific epicenters were identified via 1,000-repetition spatial permutation tests. The color bars indicate the percentage of patients (%) in whom a given cortical region emerged as a statistically significant FC (red, left) or SC (blue, right) disease epicenter. T2DM, type 2 diabetes mellitus; FC, functional connectivity; SC, structural connectivity; LOOCV, leave-one-out cross-validation.
